# Challenges in assessing the immunization status of adults in Germany—lessons from a population-based VACCELERATE survey on polio vaccination

**DOI:** 10.1007/s15010-024-02296-9

**Published:** 2024-05-28

**Authors:** Julia A. Nacov, Jannik Stemler, Jon Salmanton-García, Louise M. Cremer, Markus Zeitlinger, Patrick W. G. Mallon, Zoi Dorothea Pana, Heinz-Josef Schmitt, Oliver A. Cornely, On behalf of the VACCELERATE Consortium

**Affiliations:** 1 Faculty of Medicine, Institute of Translational Research, University of CologneUniversity Hospital CologneCologne Excellence Cluster On Cellular Stress Responses in Aging-Associated Diseases (CECAD), Herderstr. 52-54, 50931 Cologne, Germany; 2 Faculty of Medicine, Department I of Internal Medicine, Excellence Center for Medical Mycology (ECMM), University of Cologne, and University Hospital Cologne, Cologne, Germany; 3 German Centre for Infection Research (DZIF), Partner Site Bonn-Cologne, Cologne, Germany; 4 Faculty of Medicine, University of Cologne, University Hospital Cologne, Clinical Trials Centre Cologne (ZKS Köln), Cologne, Germany; 5 Department of Clinical Pharmacology, Medical University of Vienna, Vienna, Austria; 6 School of Medicine, University College Dublin, Centre for Experimental Pathogen Host Research, Dublin, Ireland; 7 Medical School, European University Cyprus (EUC), Nicosia, Cyprus

**Keywords:** Pandemic preparedness, Vaccine preventable, Vaccination coverage, Public health, Surveillance, Infection control

## Abstract

**Purpose:**

Considering the re-emergence of poliomyelitis (PM) in non-endemic regions, it becomes apparent that vaccine preventable diseases can rapidly develop epi- or even pandemic potential. Evaluation of the current vaccination status is required to inform patients, health care providers and policy makers about vaccination gaps.

**Methods:**

Between October 28 2022 and November 23 2022, 5,989 adults from the VACCELEREATE Volunteer Registry completed an electronic case report form on their previous PM vaccine doses including number, types/-valencies and the time of administration based on their vaccination records. A uni-/multivariable regression analysis was performed to assess associations in participant characteristics and immunization status.

**Results:**

Among German volunteers (n = 5,449), complete PM immunization schedule was found in 1,981 (36%) participants. Uncertain immunization, due to unknown previous PM vaccination (*n* = 313, 6%), number of doses (*n* = 497, 9%), types/-valencies (*n* = 1,233, 23%) or incoherent immunization schedule (*n* = 149, 3%) was found in 40% (*n* = 2,192). Out of 1,276 (23%) participants who reported an incomplete immunization schedule, 62 (1%) never received any PM vaccine. A total of 5,074 (93%) volunteers reported having been vaccinated at least once and 2,087 (38%) indicated that they received vaccination within the last ten years. Female sex, younger age, as well as availability of first vaccination record were characteristics significantly associated with complete immunization (*p* < 0.001).

**Conclusion:**

Full PM immunization schedule was low and status frequently classified as uncertain due to lack of details on administered doses. There is an obviousneed for improved recording to enable long-term access to detailed vaccination history in the absence of a centralized immunization register.

**Supplementary Information:**

The online version contains supplementary material available at 10.1007/s15010-024-02296-9.

## Introduction

The coronavirus disease 2019 (COVID-19) pandemic has shown the global impact of emerging infectious diseases, including the burden on healthcare systems, socio-psychological implications, and economic damage [1, 2]. The global response to public health emergencies derives from a broad range of instruments implemented by means of pandemic preparedness [2]. Various response strategies, including masking, quarantining, testing and other containment efforts achieved to reduce burden and spread of COVID-19, but in particular the introduction of effective vaccines had a significant impact on reducing hospitalizations and mortality [2, 3]. Adequate and reliable documentation of administered doses is crucial not only with respect to vaccine surveillance and monitoring, but also for individual knowledge, and safety [4–6].

Poliomyelitis (PM) is a highly contagious infectious disease caused by poliovirus (PV), a non-enveloped ribonucleic acid (RNA) enterovirus, classified as member of the *picornaviridae* family [7]. It encompasses three distinct serotypes and is able to affect motor neurons of the spinal cord and brainstem, which may result in irreversible paralysis and even death [8].

Since the World Health Assembly launched the global PM eradication plan in 1988, when over 350.000 PM cases were diagnosed across 125 countries, considerable progress has been achieved [9, 10]. Most regions were certified free of wildtype poliovirus (WPV) as result of global immunization programs and mass vaccination campaigns [9, 11]. However, WPV type 1 is still circulating in Afghanistan and circulating vaccine-derived polioviruses (cVDPV), particularly type 1 and 2, are emerging in Pakistan and Ukraine, even causing outbreaks [9, 12, 13]. In July 2022, one case of PM with paralysis related to cVDPV type 2 was confirmed in an unvaccinated adult in the State of New York [14]. Moreover, cVDPV type 2 in London, United Kingdom (UK) sewage samples raised public health concerns [15]. Considering this re-emergence of PM in non-endemic regions, it becomes apparent that vaccine preventable diseases other than COVID-19, and even those assumed to be largely controlled, can rapidly develop epi- or even pandemic potential.

While PM vaccination rates in children are monitored by the RKI (German federal government agency and research institute for disease control and prevention) and ASHIP (Association of Statutory Health Insurance Physicians) in Germany, the last evaluation in adults occurred in 2013 as part of the German Health Interview and Examination Survey for Adults (DEGS1) [16, 17]. Current vaccination coverage among the German population may differ largely from these records due to vaccine hesitancy, increasing migration from Middle East and Ukraine and non-harmonized European vaccination schedules [5, 18–20].

We therefore performed this population-based survey to give insights into the vaccination coverage for PM in the German population and identify knowledge gaps within the monitoring and recording system.

## Methods

To assess the PM vaccination status in adults, the VACCELERATE Volunteer Registry (VR) was consulted. VACCELERATE is a European Commission funded consortium dedicated to vaccine research, that runs a VR to promote clinical studies and citizen science initiatives. Volunteers can sign up via an electronic survey (https://vaccelerate.eu/volunteer-registry-2) [21]. With over 30,000 adult volunteers in 16 different European countries by October 2022, it provided a considerable number of potential participants, thus allowing a broad evaluation. The VR was approved by the Ethics Commission of Cologne University’s Faculty of Medicine (Germany) (identifier 20–1536).

All registered adults (*n* = 33,207) were invited by e-mail to complete an electronic case report form (eCRF) to collect information on past PM vaccinations including all dates, as well as administered vaccine types and valences. Children were not included as specific data regarding their vaccination rates are available from the RKI and ASHIP vaccination monitoring, assessing data from school entry examinations and vaccination services from the ASHIP in Germany [6, 22, 23]. The proportion of registered children originating from other European countries involved was comparatively small. The eCRF was made available in three different languages (English/German/Greek) as adult VACCELERATE volunteers originated mainly from Germany (*n* = 31,917), Ireland (n = 678), Austria (*n* = 194) and Cyprus (*n* = 156) at time of consultation. Basic demographic data, as well as medical history including underlying diseases were matched from their registration entries. Regional differences were analyzed by comparing geographical distribution of vaccination status by using postal codes.

To assess whether data obtained was complete, a question addressing the first entry in the oldest available vaccination certificate was included. To assess maintenance/completeness of vaccination records we compared year of birth and year of first entry in the oldest available vaccination card.

The eCRF provided a fill in aid, including label names of all PM vaccines that had been available in the four countries from 1955 onwards. Additionally, explanations on vaccine types/-valencies were included and participants were able to reply by e-mail to send inquiries in case of uncertainties. Incoming replies were processed on a daily basis during the study period.

To directly address the issue of potential vaccination gaps, automated evaluation of vaccination status was provided after eCRF was completed. “Complete PM immunization status” was defined as full primary series comprising three trivalent doses and at least one booster vaccination (Supplementary Fig. 1) [24, 25]. In addition to that specific populations with increased risk of exposure to poliovirus, e.g. healthcare workers are recommended to receive a booster dose of inactivated poliovirus (IPV) vaccine or an IPV-containing vaccine every 10 years [24]. Based on entries, participants received preprogrammed replies to primarily inform them in case of incomplete immunization, defined as three or less trivalent (or equivalent mono- or bivalent) vaccines. If classification was not possible, vaccination status was classified as uncertain.

Vaccination status and primary series (basic immunization) was classified as complete, uncertain or incomplete. To further assess the group with “uncertain vaccination status”, additional analyses regarding underlying causes for uncertainty and probability for completeness despite minor or major inconsistencies were performed. To assess the probability for completeness, three different criteria including number of doses, year of application of individual doses, as well as the coherence of recommended vaccination schedules were considered as detailed below. If one or more criteria were met, uncertain vaccination status was categorized as probably complete.

To our knowledge, formerly recommended PM vaccination schedules consisted of up to six vaccines including primary series (i.e., former German Democratic Republic (GDR) vaccination schedule: three monovalent oral attenuated poliomyelitis vaccine (OPV) doses against each serotype I-III, followed by two trivalent doses) and a booster dose [24]. Therefore, one criterion for probable completeness was defined as ≥ 6 doses regardless of otherwise lacking and/or incoherent data. The second criterion, addressing the year of vaccination, was based upon the Standing Committee on Vaccination (STIKO) of the Federal Republic of Germany (FRG) recommending the sole use of trivalent inactivated poliomyelitis vaccine (IPV) to prevent cVDPV from 1998 [24]. It was assumed that all vaccines received after 1998 were IPV. If four trivalent doses were achieved, uncertain immunization was categorized as probably complete. As vaccines of different types and valences were eventually combined depending on time and place of administration, we valued mono-/bivalent vaccines differently than trivalent doses (i.e., three monovalent doses equal one trivalent dose) to assess the coherence to recommended vaccination schedules [24]. Besides the GDR vaccination schedule, other former recommended vaccination schemes including the FRG vaccination scheme with three trivalent doses for primary series were considered according to archived vaccination schedule tables by the STIKO [24, 26]. For this sub analysis, the pattern of reported vaccines was holistically assessed. If an immunization schedule was correctly pursued, but minor specifications were lacking, it was assumed that the vaccination schedule was completed as recommended (e.g., complete primary series, followed by PM vaccine of unknown type/valency) and therefore categorized as probably complete.

To provide a scale projection specific for Germany, as well as assessment as to the representativity in terms of sex- and age-related, as well as regional distribution of the cohort as compared to the general population, the most recently available number of registered inhabitants by sex, age groups and federal states of registered inhabitants according to the federal Statistical Office from December 31 2021 was considered [27].Statistical analysis was performed using SPSS Statistics Version 25.0.0.0 (IBM Corp). A uni- and multivariable regression analysis was performed to assess significant associations in patient characteristics and incomplete and/or uncertain vaccination status as compared to complete immunization. Report of all the individual P values and confidence intervals was done, with no mathematical correction for multiple comparisons. The significance level was set at a P value < 0.05.

## Results

Between October 28 2022 and November 23 2022, 5.630 of 33.207 (17%) invited volunteers completed the eCRF with most participants originating from Germany (n = 5.449, 96·8%), followed by Ireland (n = 87, 1·5%) and Austria (n = 49, 0·9%).

As the number of participants from countries other than Germany was comparatively low and not representative, respective data was not considered and included in the evaluation.

**Fig. 1:**
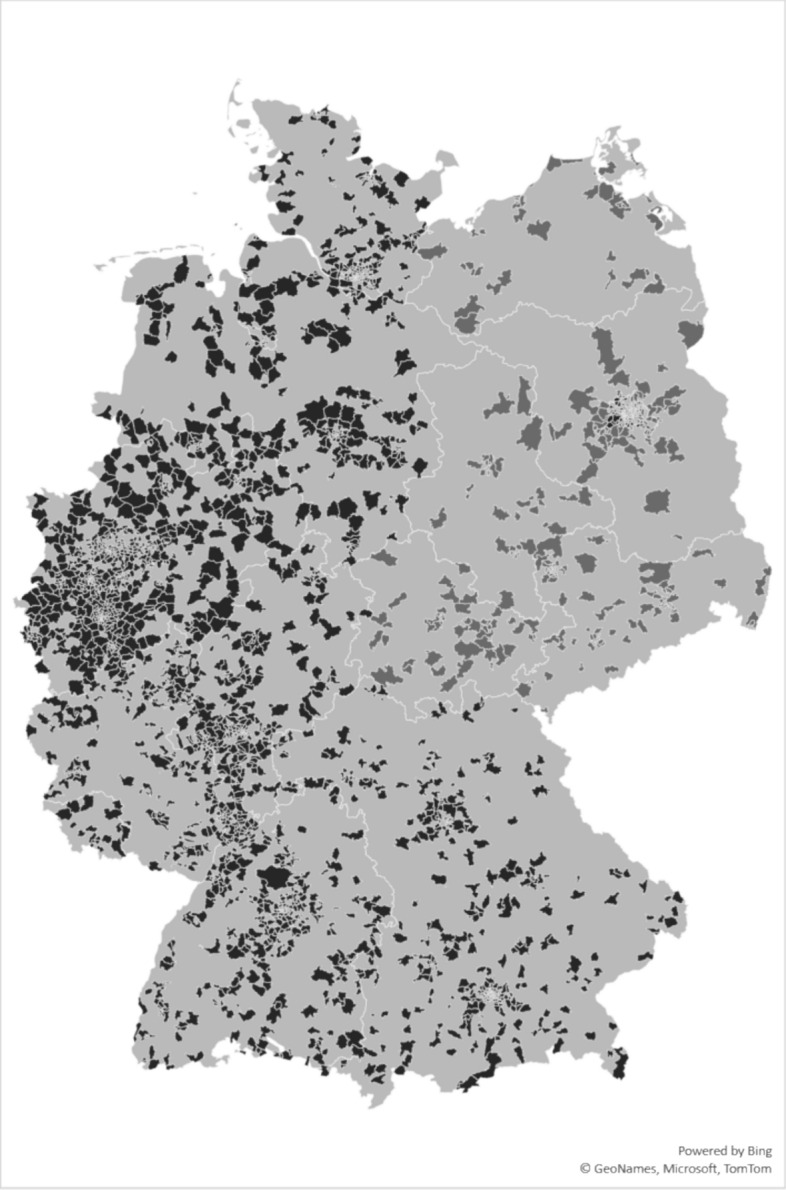
Regional Distribution of Participants

Within Germany, 3,193 (58.6%) women and 2,231 (40.9%) men provided information on their PM vaccinations. Median year of birth was 1975 (IQR 1964–1986). Detailed participant characteristics are given in Table 1, and geographical distribution in Fig. 1.Compared to the general population in Germany, the regional distribution of participants in our study was proportional to the number of inhabitants per federal state in the general population in Germany, except for overrepresentation of participants from North-Rhine-Westphalia and underrepresentation of participants from Lower Saxony. Nationwide, only the age group 40–60 years was overrepresented. Regarding sex, there was a higher proportion of women in our cohort compared to the general population in Germany (51% women vs. 49% men).

**Table 1:** Participant Characteristics and Underlying Diseases

	Overall		Completeness Primary Series for Poliomyelitis^a^						Completeness Routine Vaccination Schedule for Poliomyelitis^b^					
			Incomplete Primary Series		Uncertain Primary Series		Complete Primary Series		Incomplete Vaccination		Uncertain Vaccination		Complete Vaccination	
n** = **	5449		801		1975		2673		1276		2192		1981	
	n	%	n	%	n	%	n	%	n	%	n	%	n	%
Birth Year	1975 (1964–1986) [1932–2003]		1966 (1957–1979) [1933–2003]		1976 (1963–1985) [1936–2003]		1977 (1966–1989) [1932–2003]		1968 (1959–1983) [1933–2003]		1978 (1965–1986) [1936–2003]		1975 (1966–1988) [1932–2003]	
Gender														
Female	3193	58.6%	396	49.4%	1135	57.5%	1662	62.2%	669	52.4%	1269	57.9%	1255	63.4%
Male	2231	40.9%	404	50.4%	827	41.9%	1000	37.4%	603	47.3%	909	41.5%	719	36.3%
Non-binary	12	0.2%	1	0.1%	7	0.4%	4	0.1%	4	0.3%	6	0.3%	2	0.1%
Not Reported	13	0.2%	0	0.0%	6	0.3%	7	0.3%	0	0.0%	8	0.4%	5	0.3%
Underlying Conditions														
High Blood Pressure	788	14.5%	169	21.1%	297	15.0%	322	12.0%	235	18.4%	301	13.7%	252	12.7%
Coronary Heart Disease	97	1.8%	31	3.9%	44	2.2%	22	0.8%	41	3.2%	41	1.9%	15	0.8%
Heart Failure	54	1.0%	13	1.6%	27	1.4%	14	0.5%	19	1.5%	28	1.3%	7	0.4%
Asthma/COPD/Chronic Bronchitis/Emphysema	443	8.1%	82	10.2%	173	8.8%	188	7.0%	114	8.9%	180	8.2%	149	7.5%
Chronic Hepatitis B or C	8	0.1%	2	0.2%	4	0.2%	2	0.1%	4	0.3%	2	0.1%	2	0.1%
Chronic Non-Infectious Liver Disease	28	0.5%	5	0.6%	10	0.5%	13	0.5%	9	0.7%	9	0.4%	10	0.5%
Chronic Kidney Disease	34	0.6%	10	1.2%	13	0.7%	11	0.4%	9	0.7%	17	0.8%	8	0.4%
> 20 kg Overweight	560	10.3%	99	12.4%	236	11.9%	225	8.4%	149	11.7%	239	10.9%	172	8.7%
Diabetes Mellitus	165	3.0%	46	5.7%	75	3.8%	44	1.6%	60	4.7%	69	3.1%	36	1.8%
HIV	52	1.0%	13	1.6%	21	1.1%	18	0.7%	20	1.6%	18	0.8%	14	0.7%
Active Cancer < 2 Years	72	1.3%	14	1.7%	31	1.6%	27	1.0%	20	1.6%	36	1.6%	16	0.8%
Epilepsy	33	0.6%	7	0.9%	11	0.6%	15	0.6%	9	0.7%	14	0.6%	10	0.5%
Chronic Illness of Stomach/Intestines	49	0.9%	8	1.0%	21	1.1%	20	0.7%	15	1.2%	22	1.0%	12	0.6%
Musculoskeletal System Disease	62	1.1%	12	1.5%	32	1.6%	18	0.7%	16	1.3%	31	1.4%	15	0.8%
Mental Illness	299	5.5%	53	6.6%	111	5.6%	135	5.1%	78	6.1%	129	5.9%	92	4.6%
History of Stroke	32	0.6%	10	1.2%	9	0.5%	13	0.5%	14	1.1%	9	0.4%	9	0.5%
Other Diseases (Not Specified)	1247	22.9%	187	23.3%	473	23.9%	587	22.0%	295	23.1%	519	23.7%	433	21.9%
Maintenance of First Vaccination Certificate	3205	58.8%	140	17.5%	1028	52.1%	2037	76.2%	365	28.6%	1284	58.6%	1556	78.5%

^*a*^> 3 Trivalent Vaccine Doses for Poliomyelitis or Mono-/Bivalent Equivalent Doses

^*b*^Basic Immunization/Primary Series and One Booster Vaccination for Poliomyelitis

Details on demographic and regional representativeness of the cohort are given in the web-only supplement (Supplementary Tables 4 and 5).

### Vaccination status

Although the majority of participants reported having received five (*n* = 1,009; 18.5%), followed by four (*n* = 805; 14.8%) PM vaccine doses, complete vaccination status was found in 1,981 (36.4%) individuals only.

In 1,382 (25.4%) participants, vaccination status was uncertain despite receipt of ≥ 4 doses, as vaccine types and valency were either insufficiently specified (*n* = 1,233; 22.6%) or the immunization schedule reported was incoherent (*n* = 149; 2.7%). A total 497 (9.1%) volunteers stated they had been vaccinated, but number and valency were unknown, and 313 (5.7%) volunteers did not know whether they had been vaccinated. This resulted in 2,192 (40.2%) of participants being classified with uncertain vaccination status.

Of 1,276 (23.4%) volunteers with incomplete immunization, 62 (4.9%) volunteers declared never having received a PM vaccine, 14.7% of volunteers had incomplete primary series, and 5,074 (93.1%) volunteers reported having been vaccinated at least once.

A scaled projection for Germany (n = 84,270,625) based on our data estimated that 19,719,326 (23.4%) German inhabitants may have incomplete vaccination.

**Fig. 2:**
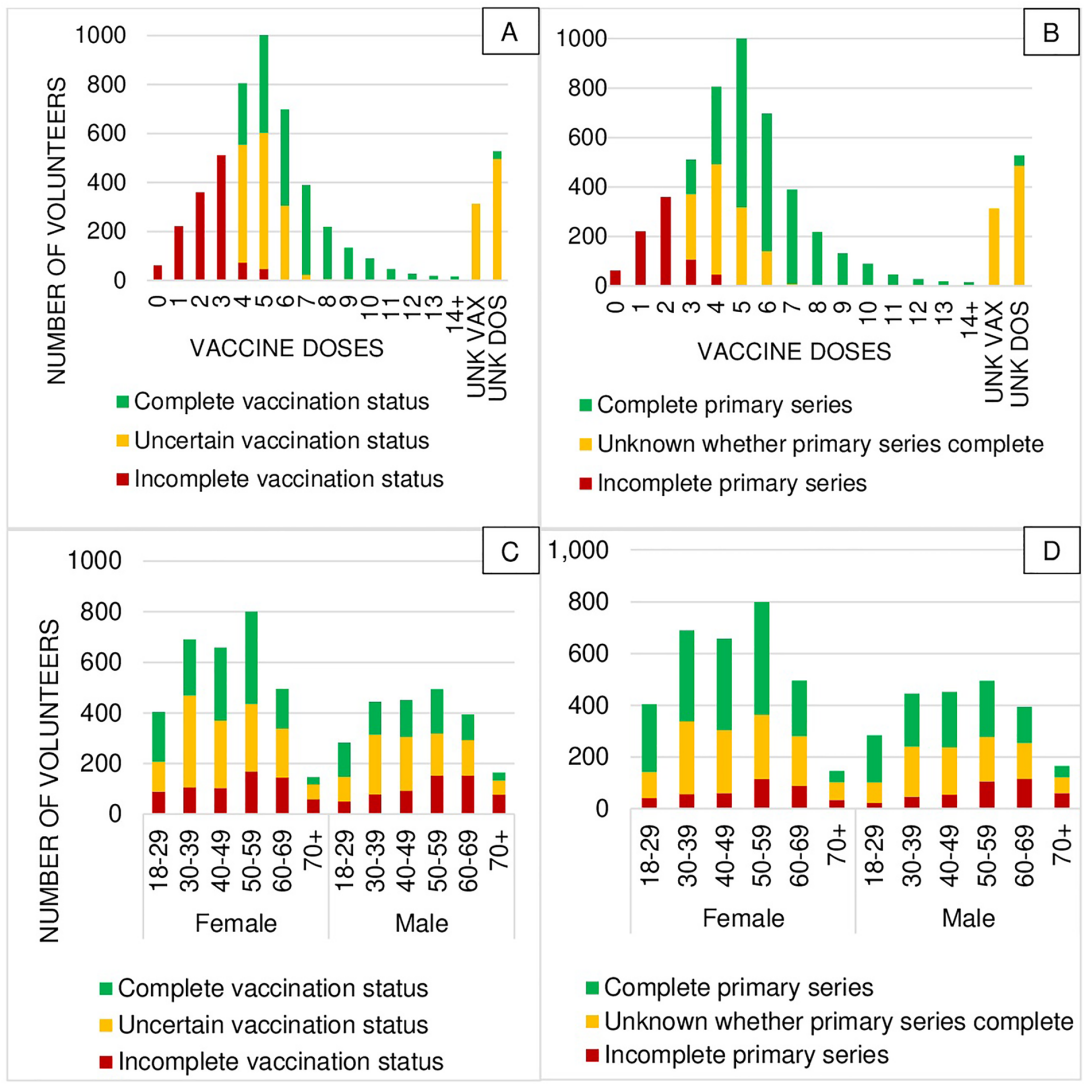
Reported Poliovirus Vaccination status (n = 5424) – Distribution by **A** Vaccination Status, **B** Primary Series, **C** and **D** additionally by Age Range and Sex. UNK VAX, unknown whether vaccinated; UNK DOS, vaccinated with an unknown number of doses

For details on age, sex, reported doses for primary series and vaccination status see Fig. 2.

### Regression analysis

#### Certificate of vaccination

Volunteers with complete vaccination status significantly more often had access to their first vaccination certificate (78.5%), when compared to those with uncertain (58.6%) or incomplete vaccination history (28.6%; *p* < 0.001). Results were similar for documented complete primary series. Median year of birth and first entry in a vaccination card was close, i.e. 1975 and 1978, in those reporting complete vaccination status. That interval was significantly larger in those with incomplete vaccination status (1968 and 1992; *p* < 0.001).

#### Demographics

Year of oldest available vaccination card equaled year of birth in 582 (84.2%) participants between 18 and 29 years, and in 45 (14.5%) participants  ≥ 70 years (Supplementary Table 2). Increasing age was associated with higher likelihood of uncertain/incomplete vaccination status and primary series in uni-, as well as multivariable analysis (Table 2). Correspondence of year of birth and first entry in a vaccination card was significantly more frequent in female when compared to male participants (63.0% vs. 52.8%; *p* < 0.001). Female participants were more likely to have a documented complete vaccination than male participants (39.3% vs. 32.2%, *p* < 0.001). Male sex was associated with incomplete and uncertain vaccination status, as well as primary series (Table 2). Highest proportion of complete vaccination was found in female participants between 18 and 29 years (48.5%), and 50 and 59 years (45.7%). The highest proportion of incomplete vaccination status was found in male participants > 70 years (47.0%) (Fig. 1C). The same applied for primary series, but with a wider range (64.9% vs. 36.6%) compared to vaccination status (Fig. 1D).

**Table 2:** Regression Analysis

	Association with Uncertain/Incomplete Vaccination (as Compared to Complete Vaccination)									Association with Complete Primary Series^a^ (as Compared to No/Unknown Primary Series)								
	Univariable Analysis				Multivariable Analysis					Univariable Analysis					Multivariable Analysis			
	P Value	OR	95% CI		P Value	OR	95% CI		P Value		OR	95% CI		P Value		OR	95% CI	
			Lower Limit	Upper Limit			Lower Limit	Upper Limit				Lower Limit	Upper Limit				Lower Limit	Upper Limit
Age	< .001	1.014	1.010	1.018	< .001	0.985	0.980	0.990	< .001		0.979	0.975	0.983	< .001		1.010	1.004	1.016
Gender																		
Female	–	–	–	–	–	–	–	–	–		–	–	–	–		–	–	–
Male	< .001	1.340	1.195	1.503	0.001	1.241	1.087	1.417	< .001		0.753	0.675	0.840	0.011		0.837	0.730	0.960
Non-binary	0.130	3.231	0.707	14.773	0.111	3.724	0.740	18.738	0.206		0.460	0.138	1.532	0.114		0.334	0.086	1.299
Year of Last Polio Vaccination	< .001	0.973	0.969	0.977	< .001	0.970	0.965	0.975	< .001		1.038	1.033	1.042	< .001		1.040	1.035	1.045
Maintenance of First Vaccination Certificate	< .001	0.247	0.218	0.280	< .001	0.277	0.234	0.327	< .001		4.409	3.924	4.955	< .001		3.441	2.923	4.051
Underlying Conditions																		
High Blood Pressure	0.005	1.261	1.073	1.482	< .001	0.699	0.569	0.859	< .001		0.676	0.580	0.788	0.002		1.396	1.127	1.729
Coronary Heart Disease	< .001	3.188	1.833	5.544	0.210	1.524	0.789	2.942	< .001		0.298	0.185	0.481	0.191		0.666	0.362	1.225
Heart Failure	< .001	3.891	1.755	8.625	0.027	2.682	1.122	6.414	0.001		0.359	0.195	0.662	0.300		0.667	0.309	1.436
Asthma/COPD/Chronic Bronchitis/Emphysema	0.199	1.144	0.932	1.405					0.003		0.745	0.612	0.907	0.141		0.830	0.648	1.064
Chronic Hepatitis B or C	0.506	1.722	0.347	8.539					0.192		0.345	0.070	1.710					
Chronic Non-Infectious Liver Diseases	0.936	1.032	0.476	2.241					0.776		0.897	0.426	1.890					
Chronic Kidney Disease	0.122	1.871	0.845	4.140					0.055		0.493	0.240	1.014					
> 20 kg Overweight	0.003	1.332	1.103	1.609	0.493	1.087	0.856	1.381	< .001		0.667	0.558	0.797	0.356		0.892	0.700	1.137
Diabetes Mellitus	< .001	2.097	1.443	3.047	0.122	1.413	0.911	2.191	< .001		0.366	0.258	0.519	0.001		0.494	0.323	0.757
HIV	0.155	1.563	0.845	2.892					0.038		0.545	0.307	0.968	0.092		0.545	0.269	1.104
Active Cancer < 2 Years	0.013	2.024	1.158	3.538	0.415	1.309	0.685	2.502	0.049		0.618	0.382	0.998	0.438		1.295	0.673	2.491
Epilepsy	0.463	1.321	0.628	2.782					0.673		0.863	0.434	1.715					
Chronic Illness of Stomach/Intestine	0.085	1.777	0.924	3.416					0.245		0.712	0.402	1.262					
Musculoskeletal System Disease	0.047	1.808	1.008	3.242	0.662	0.846	0.398	1.795	0.002		0.420	0.242	0.728	0.904		0.954	0.444	2.048
Mental Illness	0.036	1.309	1.017	1.685	0.014	1.433	1.076	1.908	0.158		0.845	0.668	1.068					
History of Stroke	0.329	1.469	0.678	3.181					0.338		0.707	0.349	1.435					

^*a*^> 3 Trivalent Vaccine Doses for Poliomyelitis or Mono-/Bivalent Equivalent Doses

#### Underlying diseases

Within participants with incomplete vaccination status, 1,017 (80.0%) had underlying chronic disease (Table 1), of whom 38 (3.0%) reported an immunodeficiency including human immunodeficiency virus (HIV) (*n* = 19; 1.5%) or active cancer within recent two years (*n* = 19; 1.5%). Furthermore, hypertension and diabetes mellitus were significantly associated with incomplete or uncertain vaccination and primary series (Table 2).

#### Regional differences

No significant regional differences were observed for vaccination status or primary series (Supplementary Table 1). The proportion of volunteers with chronic kidney disease (1.8% vs. 0.5%; *p* < 0.001), as well as psychiatric disorders (7.9% vs. 5.4%; *p* < 0.02) were significantly higher in volunteers from eastern as compared to western German states. Also, maintenance of first vaccination certificate was more likely in volunteers from eastern states (64.3% vs. 58.2%; *p* < 0.06).

#### Timing of vaccination

**Fig. 3:**
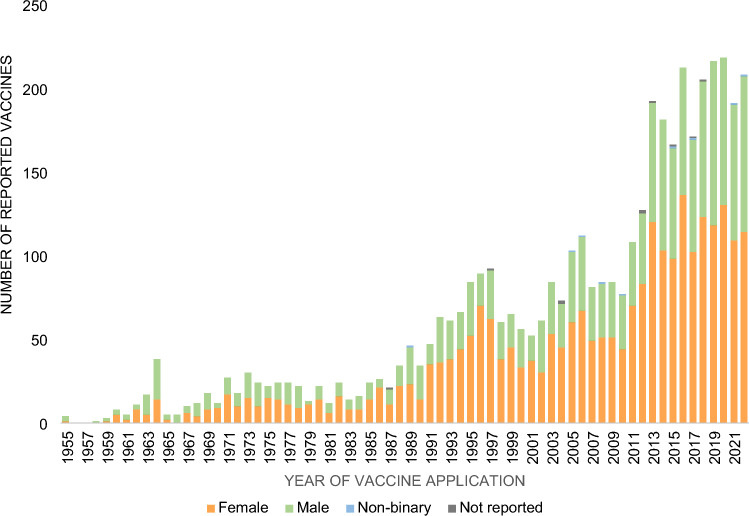
Number of Poliovirus Vaccine Doses Reported by Gender and Year

A total of 2,087 (38.3%) participants indicated that they received a PM vaccine within the last 10 years. Most vaccines were obtained in 2020 (*n* = 218; 5.1%), closely followed by 2019 (*n* = 216; 5.0%). No increase was observed for 2022 as compared to prior four years (Fig. 2). Longer interval since last vaccination (> 1 year) was associated with incomplete primary series and vaccination status (Table 2). While between 1955 and 1991, vaccination rates were overall higher in men compared to women, vaccination rates were consistently higher in women from 1991 (Fig. 3). In 1958 and 1966 only men reported on receipt of PM vaccination.

### Sub-analysis of cohort with uncertain vaccination status

Sub-analysis of vaccines reported by volunteers with uncertain vaccination status revealed that out of 2,192 participants, 423 (19.3%) were probably complete (Supplementary Fig. 2). Added to those classified as complete (*n* = 1,981), a total of 2,404 (44.1%) volunteers would have complete vaccination status. Underlying causes for uncertain vaccination status were identified as unknown vaccine types and/or valences (56.3%), unknown number of doses (22.7%), unknown PM vaccination (14.3%) and incoherent vaccination scheme (6.8%). Most frequently applied criterion was number of doses (*n* = 365; 85.1%), followed by coherence of vaccination schedule (*n* = 58; 13.5%) and year of application (*n* = 6; 1.4%).

## Discussion

Using poliomyelitis as a precedent, this investigation shows that current immunization recording in Germany is not fit for the next pandemic. While 23% of volunteers had an incomplete vaccination status according to the recommended schedule besides fully available individual vaccination records, more than 40% of participants were classified with uncertain vaccination status, mostly due to the lack of specific details on previously administered doses.

For vaccines less established than those against PM, or less often part of a combination vaccine, rates must be assumed worse [9, 24]. The rates found in our study appear even direr when seen against a background of successful global mass PM vaccination campaigning, and considering that adult volunteers in the VACCELERATE registry may likely more interested in the topic [9, 21].

Pandemic preparedness comprises knowledge of vaccination gaps at a population-level [2]. The (re-)emergence of highly contagious and vaccine preventable pathogens demands for broad, rapid and specific knowledge of previously administered vaccine doses to target those with incomplete vaccination schedules [2, 3]. Sufficient vaccination coverage is required to induce protective population immunity and prevent outbreaks [28]. In the absence of a centralized vaccination registry data on individual previous immunization can only be derived from personal vaccination certificates, medical records or self-reports [5, 18, 20].

With an increased level of migration on the one hand, and growing vaccine hesitancy on the other hand, recent isolation of PV appears even more alarming behind the background that the greatest risk for re-establishment arises from the juxtaposition of un-/under immunized- and large populations vaccinated with IPV-only schemes [14, 18, 20, 29]. Since 1998 the Standing Committee on Vaccination (STIKO) of the Federal Republic of Germany (FRG) recommends the sole use of IPV to prevent cVDPV [24]. According to the risk assessment by the European Centre for Disease Prevention and Control (ECDC), there is a moderate risk of infection and low risk for disease in IPV-only vaccinees, while OPV vaccinees have a very low risk for both, infection and disease [29]. Low- and unvaccinated groups on the contrary have a high probability of infection and moderate risk for disease [29]. The calculated herd immunity threshold of 80 to 86% would not be reached according to our study data [28].

The last evaluation of the PM vaccination status in German adults was accomplished over ten years ago as part of the first wave of German Health Interview and Examination Survey for Adults (DEGS1) [16]. Data collected from vaccination cards and self-reports thereby revealed a lifetime prevalence for at least one PM vaccination of 85.6% [16]. In the present study, we found a slightly higher lifetime vaccination prevalence with 93.1% among registered volunteers. Recent public warnings after PV detection in non-endemic regions may have motivated people to check their PM immunization status [30]. However, our results showed no significant increase in vaccination rates in 2022 compared to the four preceding years, while most doses were recorded in 2020 at the beginning of the COVID-19 pandemic.

A 2002 PM seroprevalence study on PM in Germany including sera from 2.564 adults indicated high levels of population immunity showing neutralizing antibodies against PV type 1 in 96.8% of subjects, as well as 96.8% against type 2 and 89.6% type 3 respectively [31]. This study also evaluated regional differences in immunization status. Higher rates of sufficient PM immunization were assessed in sera from individuals from Western German states [31]. In our study, no significant differences in reported PM vaccination status were found in volunteers from Western versus Eastern states. These geographic analyses warrant caution as volunteers may have moved between regions.

Similar to our findings, there was an association between demographics and vaccination status in DEGS1, showing higher vaccination rates in women compared to men and in younger compared to older participants [16]. Highest sex related difference in vaccination rates was found in both, DEGS1 and our study, in the 50–59 years age group. Higher vaccination rates in women were also reported for other vaccine preventable diseases and geographic locations for example influenza in the US and COVID-19 in Canada [32, 33]. As these are not childhood vaccinations like PM, sex-specific differences in health care adherence and decision-making that might have played a role there are less plausible causes regarding PM vaccination rates [32, 34].

In DEGS1, women were able to present their vaccination certificate more often [16]. Highest rates of complete vaccination records were found in women between 18 and 29 years and lowest rates in men between 60 and 69 years [16]. Our study showed similar findings. Year of birth and oldest available certificate entry was higher in female and younger participants. Not surprisingly, we found that the closer the oldest available vaccination certificate was to birth year, the more likely immunization records were complete. We assume incomplete vaccination records and increasing recall bias over time are main causes for reporting incomplete vaccination status. Each vaccine obtained in Germany is documented in a personal paper-based vaccine certificate [16]. Seasonal vaccines, e.g. influenza, are not necessarily documented here. If space for documentation is used up, individuals may obtain a new certificate. As there is no integral harmonized vaccination recording in Germany, data on previous vaccines can be lacking if a particular certificate is lost [5].Our study has several limitations. It is questionable whether our sample is representative as participants of the VACCELERATE Volunteer Registry (VR) may be more interested in vaccination, suggesting that the dark figure of incomplete vaccination might be even higher. While the VR was promoted in refugee reception- and health department centres, the immigrant status was not assessed in the present investigation [21]. Nevertheless, a projection for Germany based upon our data would still result in a considerable number of at least 19.719.326 (23.4%) individuals with incomplete vaccination status potentially at risk when exposed to PV [27]. Hypothetically, seropositivity might be higher taking the high number of volunteers with any vaccination against PM into account. It is important to note that this study is limited by the fact that serological analyses were not included. The study was not meant to assess seroprevalence of protection against PV but collect available data on prior administered vaccine doses to identify potential vaccination gaps at a population-level and inform participants in case of incomplete vaccination schedule.

Further limitations consist of recall and/or reporting bias as vaccines were often administered many years ago, and data transfer from a paper-based vaccination certificate into an eCRF can be erroneous. Future PM vaccination status evaluations may use technical assistance for automated reading of vaccination certificates, such as the Vacuna® app employing image recognition [35].

Ideally, vaccinations were recorded electronically in a centralized registry to allow for permanent and online-availability, and for international harmonization as individually introduced for COVID-19 vaccination during the pandemic [4]. In daily routine, missing vaccination records cause uncertainty among treating physicians as well as time-consuming investigations by medical staff [25]. The involvement of electronic tools would allow verification of self-reported data [35]. Social structures between individual participants and/or common households were not assessed within this investigation to assess the potential impact on reported data with special regard to gender-specific differences.

In conclusion, PM vaccination status was difficult to assess due to absent or incomplete records. The current paper-based vaccination recording system in Germany fails to inform the individual citizen and public health authorities. Further investigations including serological analyses adding to the data collected in this study are needed to elucidate the population PM immunization status, especially considering vulnerable patient groups with uncertain or incomplete vaccination history.

### Supplementary Information

Below is the link to the electronic supplementary material.

Supplementary file1 (DOCX 161 KB)

## Data Availability

Data available on request from the authors. The data that support the findings of this study are available from the corresponding author, OAC, upon reasonable request. Basic, Share upon Request.
