# Supplementary material for: Challenges in assessing the immunization status of adults in Germany—lessons from a population-based VACCELERATE survey on polio vaccination
Source: Infection. 2024 May 28;52(4):1563–74. doi: 10.1007/s15010-024-02296-9 (PMC11289296; doi:10.1007/s15010-024-02296-9)
Supplement: Supplementary file 1 — Supplementary file1 (DOCX 161 KB) [file 15010_2024_2296_MOESM1_ESM.docx]

**Supplementary Material**

**Table of Contents**

1 Supplementary Tables

Supplementary Table 1. Eastern vs. Western Germany by Volunteer Characteristics

Supplementary Table 2. Completeness of Vaccination Records by Age Range / Gender

Supplementary Table 3. Vaccination Status by Age Range / Gender

Supplementary Table 4. Regional Distribution of Participants in Germany by Federal States as Compared to the General Population

Supplementary Table 5. Distribution of Participants by Age Groups Compared to the General Population in Germany

2 Supplementary Figures

Supplementary Figure 1. Vaccination Status Classification in the VACCELERATE Volunteer Registry

Supplementary Figure 2. Sub Analysis Uncertain Vaccination Status – Probability for Completeness

# Supplementary Tables

# Supplementary Table 1. Eastern vs. Western Germany by Volunteer Characteristics

|  | Western Germany | | Eastern Germany | | p value |
| --- | --- | --- | --- | --- | --- |
|  | n | % | n | % |  |
| Birth Year | 1974 (1963-1986) [1932-2003] | | 1978 (1966-1987) [1941-2003] | | **0.022** |
| Sex |  |  |  |  | 0.751 |
| Female | 2798 | 58.8% | 325 | 58.0% |  |
| Male | 1952 | 41.0% | 233 | 41.6% |  |
| Non-binary | 10 | 0.2% | 2 | 0.4% |  |
| Underlying Conditions |  |  |  |  |  |
| High Blood Pressure | 700 | 14.7% | 88 | 15.7% | 0.525 |
| Coronary Heart Disease/History of Heart Attack | 86 | 1.8% | 11 | 2.0% | 0.792 |
| Heart Failure | 47 | 1.0% | 7 | 1.3% | 0.558 |
| Asthma/COPD/Chronic Bronchitis or Emphysema | 390 | 8.2% | 53 | 9.5% | 0.303 |
| Chronic Hepatitis B or C | 8 | 0.2% | 0 | 0.0% | 0.332 |
| Chronic Non-Infectious Liver Disease | 25 | 0.5% | 3 | 0.5% | 0.974 |
| Chronic Kidney Disease | 24 | 0.5% | 10 | 1.8% | **<0.001** |
| >20 kg Overweight | 497 | 10.4% | 63 | 11.3% | 0.555 |
| Diabetes Mellitus | 143 | 3.0% | 22 | 3.9% | 0.233 |
| HIV | 49 | 1.0% | 3 | 0.5% | 0.261 |
| Active Cancer <2 Years | 67 | 1.4% | 5 | 0.9% | 0.319 |
| Epilepsy | 31 | 0.7% | 2 | 0.4% | 0.402 |
| Chronic Illness of Stomach/Intestine | 45 | 0.9% | 4 | 0.7% | 0.588 |
| Musculoskeletal System Disease | 58 | 1.2% | 4 | 0.7% | 0.293 |
| Mental Illness | 255 | 5.4% | 44 | 7.9% | **0.015** |
| History of Stroke | 30 | 0.6% | 2 | 0.4% | 0.429 |
| Primary Series (Basic Immunization) |  |  |  |  | 0.286 |
| No Primary Series | 711 | 14.9% | 73 | 13.0% |  |
| Unknown whether Primary Series | 1706 | 35.8% | 217 | 38.8% |  |
| Complete Primary Series | 2343 | 49.2% | 270 | 48.2% |  |
| Vaccination Schedule Completeness |  |  |  |  | 0.397 |
| Incomplete Vaccination | 1129 | 23.7% | 119 | 21.3% |  |
| Uncertain Vaccination | 1900 | 39.9% | 235 | 42.0% |  |
| Complete Vaccination | 1731 | 36.4% | 206 | 36.8% |  |
| Maintenance of First Vaccination Certificate | 2770 | 58.2% | 360 | 64.3% | **0.006** |

# Supplementary Table 2. Completeness of Vaccination Records by Age Range / Gender

|  | Correspondence year of birth and year of oldest available vaccination card | | | |
| --- | --- | --- | --- | --- |
|  | no | | yes | |
| Age range | n | % | n | % |
| 18-29 | 109 | 15.8 | 582 | 84,2 |
| 30-39 | 229 | 20.1 | 911 | 79,9 |
| 40-49 | 316 | 28.4 | 797 | 71,6 |
| 50-59 | 647 | 49.8 | 653 | 50,2 |
| 60-69 | 677 | 75.9 | 215 | 24,1 |
| 70+ | 265 | 85.5 | 45 | 14,5 |
| Sex |  |  |  |  |
| Female | 1155 | 37.0 | 1968 | 63,0 |
| Male | 1031 | 47.2 | 1154 | 52,8 |
| Non-binary | 4 | 33.3 | 8 | 66,7 |

# Supplementary Table 3. Vaccination Status by Age Range / Gender

|  | | Incomplete vaccination status  n=1,276 | Uncertain vaccination status  n=2,192 | Complete vaccination status  n=1,981 | |
| --- | --- | --- | --- | --- | --- |
| Female | 18-29 | 88 | 120 | 196 |  |
|  | 30-39 | 106 | 363 | 221 |  |
|  | 40-49 | 103 | 267 | 288 |  |
|  | 50-59 | 168 | 267 | 365 |  |
|  | 60-69 | 145 | 193 | 157 |  |
|  | 70+ | 59 | 59 | 28 |  |
|  | Not reported | 0 | 0 | 0 |  |
| Male | 18-29 | 50 | 98 | 135 |  |
|  | 30-39 | 78 | 236 | 131 |  |
|  | 40-49 | 93 | 212 | 146 |  |
|  | 50-59 | 152 | 167 | 175 |  |
|  | 60-69 | 153 | 140 | 101 |  |
|  | 70+ | 77 | 56 | 31 |  |
|  | Not reported | 0 | 0 | 0 |  |
| Non-binary | 18-29 | 2 | 1 | 1 |  |
|  | 30-39 | 0 | 2 | 0 |  |
|  | 40-49 | 2 | 1 | 0 |  |
|  | 50-59 | 0 | 0 | 1 |  |
|  | 60-69 | 0 | 2 | 0 |  |
|  | 70+ | 0 | 0 | 0 |  |
|  | Not reported | 0 | 0 | 0 |  |
| Not reported | 18-29 | 0 | 2 | 0 |  |
|  | 30-39 | 0 | 2 | 1 |  |
|  | 40-49 | 0 | 1 | 0 |  |
|  | 50-59 | 0 | 1 | 4 |  |
|  | 60-69 | 0 | 1 | 0 |  |
|  | 70+ | 0 | 0 | 0 |  |
|  | Not reported | 0 | 1 | 0 |  |

# Supplementary Table 4. Regional Distribution of Participants in Germany by Federal States as Compared to the General Population

| **State** | **State code** | **Inhabitants** | **Inhabitant %** | **Volunteers** | **Volunteer %** | **Difference** |  |  |
| --- | --- | --- | --- | --- | --- | --- | --- | --- |
| **Baden-Württemberg** | BW | 11.148.904 | 13,40% | 658 | 11,42% | -1,98% |  |  |
| **Bavaria** | BY | 13.203.592 | 15,80% | 624 | 10,83% | -4,97% |  |  |
| **Berlin** | BE | 3.689.708 | 4,40% | 295 | 5,12% | 0,72% |  |  |
| **Brandenburg** | BB | 2.546.685 | 3,10% | 139 | 2,41% | -0,69% |  |  |
| **Bremen** | HB | 663.567 | 0,80% | 30 | 0,52% | -0,28% |  |  |
| **Hamburg** | HH | 1.904.212 | 2,30% | 170 | 2,95% | 0,65% |  |  |
| **Hesse** | HE | 6.313.614 | 7,60% | 504 | 8,74% | 1,14% |  |  |
| **Lower Saxony** | NI | 8.045.829 | 9,60% | 57 | 0,99% | -8,61% |  |  |
| **Mecklenburg-Vorpommern** | MV | 1.605.259 | 1,90% | 332 | 5,76% | 3,86% |  |  |
| **North Rhine-Westphalia** | NW | 17.944.923 | 21,50% | 2227 | 38,64% | 17,14% |  |  |
| **Rhineland-Palatinate** | RP | 4.126.872 | 4,90% | 238 | 4,13% | -0,77% |  |  |
| **Saarland** | SL | 1.005.796 | 1,20% | 25 | 0,43% | -0,77% |  |  |
| **Saxony** | SN | 4.036.369 | 4,80% | 170 | 2,95% | -1,85% |  |  |
| **Saxony-Anhalt** | ST | 2.155.742 | 2,60% | 63 | 1,09% | -1,51% |  |  |
| **Schleswig-Holstein** | SH | 2.936.486 | 3,50% | 145 | 2,52% | -0,98% |  |  |
| **Thuringia** | TH | 2.099.527 | 2,50% | 87 | 1,51% | -0,99% |  |  |
| **Total** |  | 83.427.085 | 100,00% | 5.449 | 100,00% | 0,00% |  |  |

**Federal Statistical Office. Population - Number of inhabitants in Germany by relevant age group on 31 December 2021 (in millions)*

# Supplementary Table 5. Distribution of Participants by Age Groups Compared to the General Population in Germany

| ***[years]*** | **General population** | **% general population** | **% excluding <20** | **Volunteers** | **% volunteers** | **% excluding <20** | **Difference** | **Difference excluding <20** |
| --- | --- | --- | --- | --- | --- | --- | --- | --- |
| **Overall** | 84.358.845 | 100,00% |  | 5449 | 100,00% | 5414 |  |  |
| **< 20** | 15.861.073 | 18,80% |  | 35 | 0,61% |  | -18,19% |  |
| **20 - 40** | 20.636.488 | 24,50% | 30,10% | 1778 | 30,84% | 31,03% | 6,34% | 6,53% |
| **40 - 60** | 22.999.053 | 27,30% | 33,60% | 2528 | 43,85% | 44,12% | 16,55% | 16,82% |
| **60 - 80** | 18.749.279 | 22,20% | 27,40% | 1387 | 24,06% | 24,21% | 1,86% | 2,01% |
| **80 -100** | 6.088.104 | 7,20% | 8,90% | 37 | 0,64% | 0,65% | -6,56% | -6,55% |
| **> 100** | 24.848 | 0,00% | 0,00% | 0 | 0,00% | 0,00% | 0,00% | 0,00% |

**Federal Statistical Office. Population - Number of inhabitants in Germany by relevant age group on 31 December 2021 (in millions)*

# Supplementary Figures

# Supplementary Figure 1. Vaccination Status Classification in the VACCELERATE Volunteer Registry


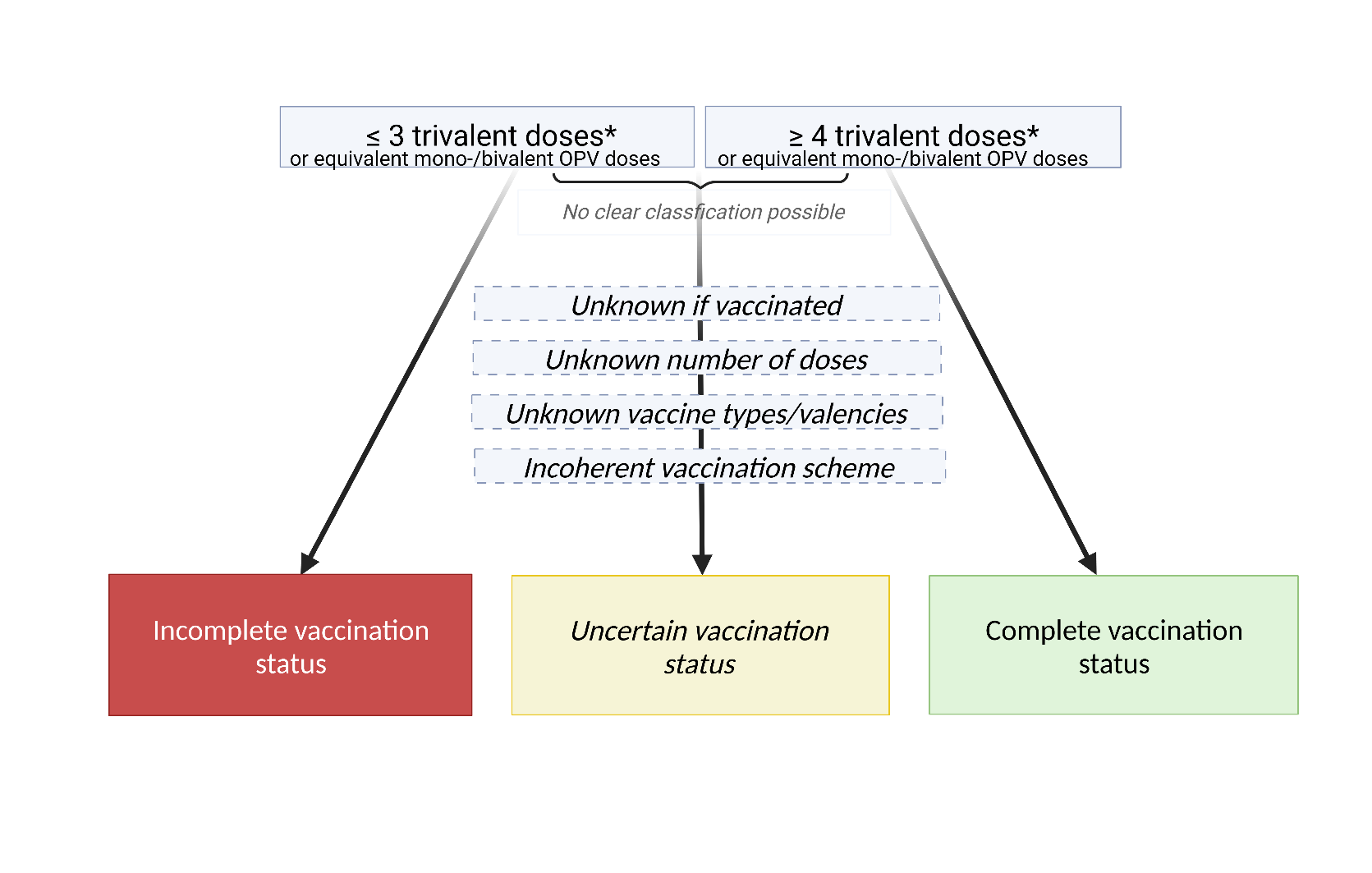


*IPV (inactivated poliovirus vaccine given by injection)/trivalent OPV (oral poliovirus vaccine)

# Supplementary Figure 2. Sub Analysis Uncertain Vaccination Status – Probability for Completeness
